# The Effects of Moxibustion on Learning and Memory and m6A RNA Methylation in APP/PS1 Mice

**DOI:** 10.1155/2022/2998301

**Published:** 2022-03-21

**Authors:** Qin Huang, Chen-yu Li, Ning Zhang, Qun Zhang, Hong-ying Li, Yuan Shen, Lu-shuang Xie, Shu-guang Yu, Qiao-feng Wu

**Affiliations:** ^1^Acupuncture and Tuina College, Chengdu University of Traditional Chinese Medicine, Chengdu, Sichuan, China; ^2^College of Basic Medicine, Chengdu University of Traditional Chinese Medicine, Chengdu, Sichuan, China

## Abstract

**Objectives:**

To study whether moxibustion can improve the learning and memory ability of APP/PS1 mice by reducing the pathological products A*β* and Tau protein via decreasing N6-methyladenosine (m6A).

**Methods:**

APP/PS1 mice were randomly divided into model group (APP/PS1) and moxibustion group (APP/PS1+Mox). C57BL/6J mice were used as a control group (Control). Learning and memory abilities were assessed by the Morris water maze. A*β*, Tau, phosphorylated Tau (p-Tau), and YTHDF1 proteins were detected in the mouse cortex and hippocampus by immunofluorescence and western blot. Altered m6A expression levels in hippocampal and cortical tissues were measured with the m6A RNA methylation quantification assay kit. RNA transcript levels of YTHDF1, METTL3, and FTO in the hippocampus and cortex were measured by q-PCR.

**Results:**

Moxibustion shortened the escape latency, increased the number of platform crossings, and increased the percentage of swimming time in the target quadrant of APP/PS1 mice. Meanwhile, moxibustion reduced the levels of A*β*, Tau, and p-Tau proteins both in the hippocampal and cortical regions of APP/PS1 mice. In addition, the total amount of m6A in the hippocampal and cortical regions of APP/PS1 mice was significantly reduced after moxibustion. The expression of YTHDF1 in the hippocampal region of APP/PS1 mice increased and that in the cortical region decreased after moxibustion treatment.

**Conclusion:**

Moxibustion improves the learning and memory abilities and reduces the deposition of A*β* and Tau protein pathological products in APP/PS1 mice. This may be related to the fact that moxibustion reduces the total amount of m6A and inhibits its binding enzyme YTHDF1 in the hippocampus and cortex of APP/PS1 mice.

## 1. Introduction

Alzheimer's disease (AD) is a progressive degenerative disease of the central nervous system characterized by progressive learning and memory impairment and cognitive dysfunction [1]. The average life expectancy of patients after being diagnosed with Alzheimer's disease is only 8–10 years, and there are currently nearly 50 million AD patients worldwide [2]. It is estimated that the number of AD patients will be 130 million by 2050 [3], undoubtedly placing a tremendous pressure and burden on families and society. Currently, there is no effective drug that can completely cure AD. Many patients ask for complementary alternative medicine, such as acupuncture and moxibustion, since they can delay the loss of learning and memory ability to some extent. Although some studies found that acupuncture and moxibustion can improve AD symptoms by regulating energy metabolism [4, 5], glial cell polarization [6], and neuroinflammation [7]. But the mechanisms have not been explored fully.

As we know, both genetic and nongenetic factors can contribute to the development of Alzheimer's disease. Epigenetics, which plays a regulatory role in A*β* accumulation and synaptic plasticity [8–11], includes DNA methylation, histone modification, and regulation of gene expression mediated by noncoding RNA molecules. Similar to DNA and proteins, the chemical structure of RNA can be modified in cells as an epigenetic mechanism to control gene expression [12]. mRNA modification by N6-methyladenosine (m6A), the most prevalent internal modification in mRNA, is an important posttranscriptional mechanism of gene regulation [13] that interacts with histone modifications [14]. Several studies in recent years have shown that m6A has many neurobiological functions in the nervous system [15, 16], such as regulating learning and memory capacity, influencing self-renewal and differentiation of neural stem cells, and participating in cerebellar development and synaptic growth. Two targeted studies of m6A and AD have shown that m6A methylation genes are significantly increased in the brains of naturally aging mice, 5XFAD model mice, APP/PS1 model mice, and elderly people. Furthermore, these different genes are mainly involved in the regulation of synaptic transmission, synaptic pruning, and ion transport [17, 18], suggesting that m6A methylation is involved in the AD process.

The aim of this study is to observe whether moxibustion can improve the learning and memory abilities of mice partially through regulating m6A methylation.

## 2. Materials and Methods

### 2.1. Mice

All experiments involving animals were approved by the Institutional Animal Care and Use Committee of Chengdu University of Traditional Chinese Medicine. Five-month-old APP/PS1 double transgenic male mice [19, 20], and age-matched wild-type C57BL/6 male mice were used. All experimental animals (30 ± 2 g) were purchased from Caven's Beagle (Su Zhou) model animal research Co. Ltd (license number: SCXK (Su) 2018–0008). Mice were housed in a light-controlled room at a temperature of 22 ± 2°C and a relative humidity of 50–70%, and they were fed a normal diet and fed ad libitum. After one week of acclimation, APP/PS1 mice were randomly divided into model groups (APP/PS1) and moxibustion groups (APP/PS1+Mox), with C57BL/6 as the control group (Control).

### 2.2. Moxibustion Treatment

According to Government Channel and Points Standard GB12346-90 of China and “The Veterinary Acupuncture of China,” the “Bai hui” (CV4) and “Yong quan” (KI1) acupoints were selected. Mice in the APP/PS1+Mox group were fixed with a homemade fixator to expose their heads and soles. Moxibustion was performed locally with homemade moxa strips (size: 0.5 cm in diameter and 18 cm in length) at 2-3 cm away from the acupoints of the APP/PS1+Mox group mice, once a day, for 30 minutes each time, and for a total of 4 courses (5 days each course) of treatment. A 2-day rest was given at the end of each treatment session. Other groups used the same fixation method but without moxibustion intervention.

### 2.3. Morris Water Maze Test

The Morris water maze (MWM) was used to assess the learning and memory abilities of the mice the day after all interventions were completed. In the MWM facility, a circular pool was divided into four quadrants by a computerized identification system to better record the animals' performance. The first quadrant was the target quadrant with a diameter of 10 cm platform. The experimental process was divided into positioning navigation and space exploration, where the positioning navigation experiment was divided into visible platform positioning navigation and a hidden platform positioning navigation experiment. During the positioning navigation experiment (first 6 days), mice were randomly placed in water in the four quadrants with their backs to the pool wall. The duration of a single session was 90 seconds. The time between entering the water and climbing onto the platform and staying there for more than 5 seconds is called the escape latency period. The learning ability of the mice was assessed by recording the escape latency and the total swimming distance each day. In the spatial exploration experiment (day 7), the platform was removed and mice were placed in the water from any quadrant away from the target quadrant, with their backs to the pool wall. The number of times the mice crossed the platform and the percentage of time they stayed in the target quadrant within 90 seconds were recorded sequentially to assess the spatial memory ability of the mice.

### 2.4. Immunofluorescence Staining

The positive expressions of A*β*, Tau, p-Tau, and YTHDF1 in hippocampal and cortical tissues were detected by immunofluorescence. Briefly, frozen slices of tissues were made after paraformaldehyde perfusion. The slices were baked in an oven at 60°C for 8 h and then repaired twice with sodium citrate repair solution for 5 min each. After washing with PBS and shaking dry, 5% BSA was closed at 37°C for 1.5 h. Afterward, the following primary antibodies were added: rabbit antiA*β*1-42 (Ab201060, Abcam), rabbit anti-Tau (AB37439-100, Multi-Science), rabbit antiphospho S199 (Ab 81268, Abcam), rabbit anti- YTHDF1 (17479-1-AP, Proteintech), for 2 h at 37°C. Then, the sections were incubated overnight at 4°C. The next day, the following secondary antibodies were added after washing with PBS: goat antirabbit IgG/Alexa Fluor (BS-0295G-AF488, Bioss), and incubated at 37°C for 1.5 h. DAPI staining solution (C1005, Beyotime) was added dropwise, and PBS was used to wash off DAPI and seal the slices after 5 min. Finally, observation was performed with a fluorescence microscope (TSC SP8, Leica).

### 2.5. Western Blot

Protein expression was determined by Western blot. Appropriate amounts of mouse cortical and hippocampal tissues were weighed and lysed in lysis buffer with intact protease inhibitors without ethylenediaminetetraacetic acid (EDTA). After lysis, the tissue supernatant was extracted from the protein sample by centrifugation at 12,000 rpm for 30 minutes in a 4°C centrifuge. The protein concentration of each sample was measured using the BCA method and diluted to the super sample concentration based on the measured protein concentration. Protein samples were separated by adding 10% SDS-PAGE gel electrophoresis and wet transfer with methanol-activated PVDF membranes. 5% milk was blocked for 1 h (room temperature) and the following primary antibodies were added: rabbit anti-A*β*1-42 (Ab201060, Abcam), rabbit anti-Tau (AB37439-100, Multi-Science), rabbit anti YTHDF1 (17479- 1-AP, Proteintech), and incubated overnight at 4°C. The PVDF membrane was washed with TBST the next day. The HRP-labeled secondary antibody (1 : 5000) was added dropwise to the PVDF membrane and incubated for 1.5 h at room temperature. At the end of the secondary antibody incubation, the membrane is rewashed with TBST. The images were exposed and developed in a chemiluminescent gel imager after adding drops of ECL (Beyotime, China), and the images were saved and analyzed using Image-Pro Plus 6.0 software.

### 2.6. Quantitative Detection of m6A RNA Methylation

To detect changes in total m6A in hippocampal and cortical tissues of each group of mice, m6A was quantified using a kit (P-9005, EpiGentek). Total RNA from hippocampal and cortical tissues was extracted with Trizol. Then the RNA concentration was quantified by measuring the OD value with a NanoDrop2000 ultramicro spectrophotometer. The ratio of A260 to A280 and the concentration value were recorded. The standard curve was plotted in Excel, and the slope values were derived from the curve equation and quantified according to the formula provided in the instructions: (m6A(ng)=sample OD − NC OD/slope, m6A%=(m6A amount(ng)/*S*)*∗*100% ).

### 2.7. Real-Time PCR (qPCR)

The primer designs for the three genes in the analyzed tissues are shown in Table 1. Total RNAs from hippocampal and cortical tissues were extracted with Trizol, and cDNA was synthesized using a reverse transcription kit (HB200901, YEASEN). The qPCR amplification reaction system was 20 *μ*L: 5×iScript Reaction Mix 4 *μ*L, iScript Reverse Transcriptase 1 *μ*L, nuclease-free water 14 *μ*L, RNA template (200 ng/*μ*L) 1ul, upstream and downstream primers 2 *μ*L each, cDNA 2 *μ*L, DEPC H_2_O 6 *μ*L. The reaction conditions were as follows: predenaturation at 95°C for 10 minutes, denaturation at 95°C for 10 seconds, annealing at 60°C for 20 seconds, extension at 72°C for 15 sec, and plate reading of 40 cycles. Melting curves were used from 72 to 95°C with the addition of 0.5°C reactions for 6S, and the plates were re-read. Finally, the q-PCR results were statistically analyzed using the 2^^−△△^Ct method.

### 2.8. Statistical Analysis

All data were expressed as mean ± standard error (Mean ± SEM). Comparisons between multiple groups were performed using one-way ANOVA followed by least significant difference (LSD). Statistical analysis was performed using the software SPSS 26.0, and the level of statistical significance was set at *P* < 0.05.

## 3. Results

### 3.1. Moxibustion Improves the Learning and Memory of APP/PS1 Mice

**Figure 1:**
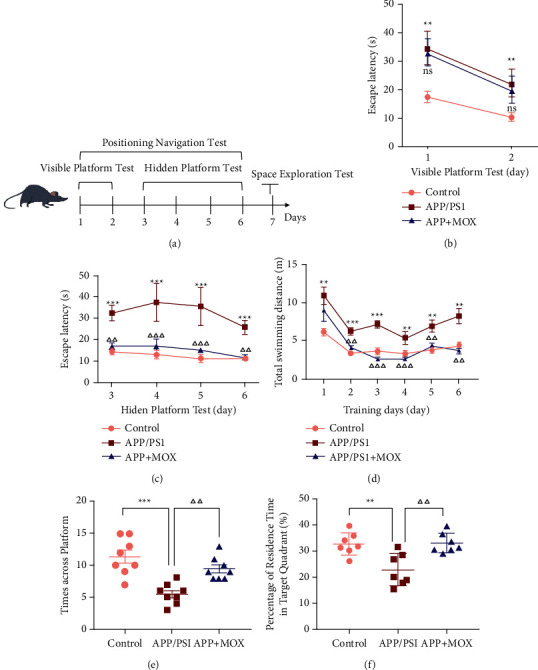
Moxibustion improves learning and memory in APP/PS1 mice. (a) Schematic diagram of the Morris water maze experiment. The trend of escape latency in the visible platform (b) and hidden platform (c) for each group. (d) Total swimming distance of different groups in the whole positioning navigation experiment. The number of times they crossed the platform (e) and the percentage of swimming time in the marked quadrant (f) in each group. ^∗^*P* < 0.05, ^∗∗^*P* < 0.01, and ^∗∗∗^*P* < 0.001 vs. control group; ^△△^*P* < 0.01 and ^△△△^*P* < 0.001 vs. APP/PS1 group.

The flow chart of the Morris water maze experiment is shown in Figure 1(a). The results of the visual platform positioning navigation experiment showed that mice in the APP/PS1 model group had increased visual platform latency on the first and second day compared to the control group (*P* < 0.01). After moxibustion, there was no difference in visual platform latency compared with the model group (Figure 1(b)). The results of the hidden platform positioning navigation experiment showed an overall decreasing trend in the escape latency of mice reaching the platform with the increase of swimming days in all groups. Compared with the control group, the escape latency of mice in all model groups was significantly increased (*P* < 0.001) but decreased with the increase of swimming days from day 4 onwards. However, after moxibustion treatment, the escape latency was significantly lower than that of the model group (*P* < 0.01) and almost the same as that of the control group (Figure 1(c)). In addition, in terms of total swimming distance, it was significantly increased in the model group (*P* < 0.05 vs control group) while significantly decreased in the moxibustion group (*P* < 0.01 vs. the model group) (Figure 1(d)).

Meanwhile, spatial exploration experiments showed that APP/PS1 mice were less active in crossing platforms than the control group (*P* < 0.001). After moxibustion, mice in the moxibustion group crossed the platform more often than the model group (*P* < 0.01) (Figure 1(e)). In addition, the percentage of time spent swimming in the target quadrant decreased in the model group mice compared with the control group (*P* < 0.01), while the percentage of time spent swimming in the target quadrant increased in the moxibustion group mice compared with the model group (*P* < 0.01) (Figure 1(f)).

### 3.2. Moxibustion Decreases A*β* Plaque in Hippocampal and Cortical Regions of APP/PS1 Mice

**Figure 2:**
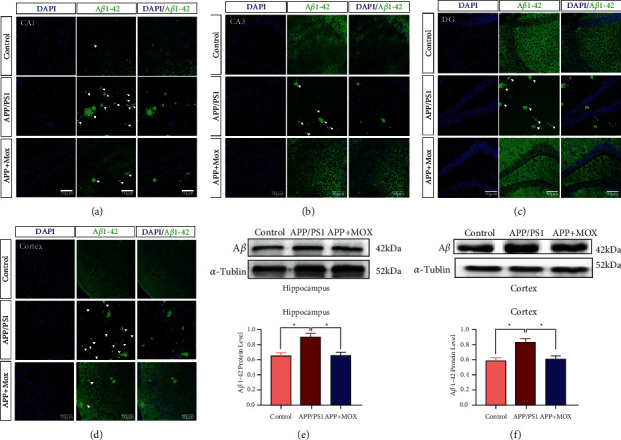
Moxibustion decreases A*β* plaque deposition both in the hippocampal and cortical regions of APP/PS1 mice. Immunofluorescence staining results of hippocampal CA1 (a), CA3 (b), DG (c), and cortical (d) area with FITC 488-conjugated A*β*1-42 antibody (green). Protein expression of A*β* in hippocampal (e) and cortex (f) tissues. White arrows indicate positive expression of A*β*1-42. Magnification in (a–d) = 200×. Scale bars in (a–d) represent 50 *μ*m. ^∗^*P* < 0.05.

Firstly, we found that the positive expression of A*β* in hippocampal CA1, CA3, DG and cortical areas of mice in the model group was significantly increased compared with the control group. After moxibustion treatment, the deposition of A*β* in hippocampal CA1, CA3, DG, and cortical regions of mice in the moxibustion group was significantly reduced compared with the model group (Figures 2(a)–2(d)). Western blotting verified the results of immunofluorescence. The relative expression of A*β* protein in the hippocampus and cerebral cortex of mice in the model group was significantly increased compared with that in the control group (*P* < 0.05). And the relative expression of A*β* protein in the hippocampus and cortical regions of mice in the moxibustion group was significantly decreased compared with the model group (*P* < 0.05) (Figures 2(e)–2(f)).

### 3.3. Moxibustion Reduces Tau and p-Tau Protein Expression in Hippocampal and Cortical Areas of APP/PS1 Mice

**Figure 3:**
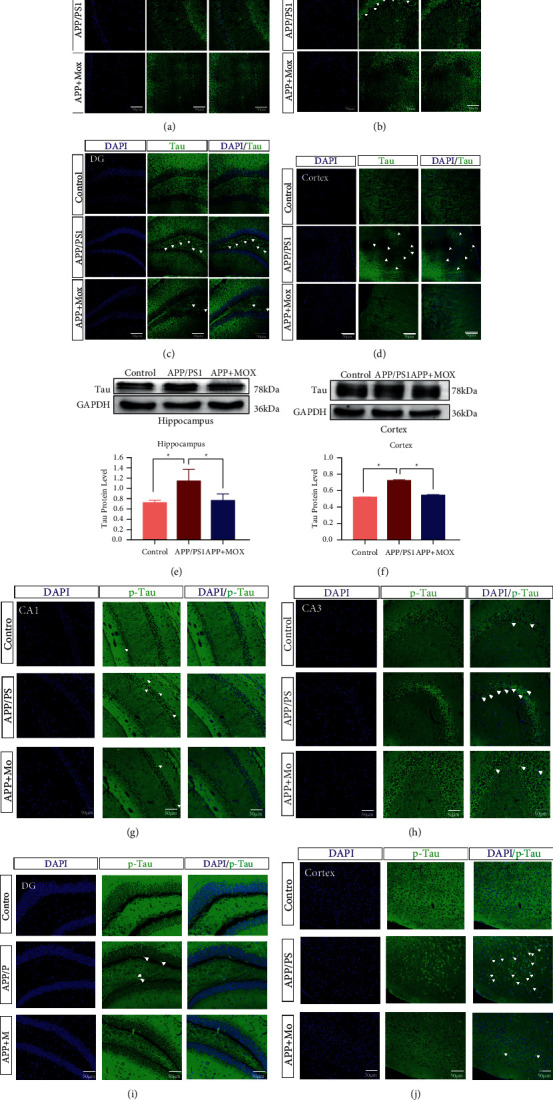
Moxibustion reduces Tau and p-Tau protein expression both in the hippocampal and cortical regions of APP/PS1 mice. Immunofluorescence staining results of hippocampal CA1 (a), CA3 (b), DG (c), and cortical (d) area with FITC 488-conjugated tau and p-tau antibody (green). Protein expression of Tau in hippocampal (e) and cortex (f) tissues. p-Tau protein immunofluorescence results in CA1 (g), CA3 (h), DG (i), and cortical regions (j). White arrows indicate positive expression of Tau and p-Tau. Magnification in (a–d) = 200×. Scale bars in (a–d) represent 50 *μ*m. ^∗^*P* < 0.05.

We also performed immunofluorescence staining for Tau in the hippocampal and cortical regions of each group of mice. Compared with the control group, the model group showed significantly increased positive expression of Tau protein in CA3, DG, and cortical areas of the hippocampus. There was no significant difference between the three groups in the CA1 area. After moxibustion treatment, the positive expression of Tau protein in CA3, DG, and cortical areas of the hippocampus was significantly reduced in the moxibustion group compared with the model group (Figures 3(a)–3(d)). In addition, the amount of Tau protein was significantly increased in hippocampal and cortical regions of mice in the model group compared with the control group (*P* < 0.05). After treatment with moxibustion, the amount of Tau protein in the hippocampal region of mice in the moxibustion group was significantly reduced compared with the model group (*P* < 0.05) (Figures 3(e)–3(f)).

We also found by immunofluorescence that the positive p-Tau protein signals in hippocampal CA1, CA3, DG area, and cortical area were increased in the model group compared with the control group, while they were significantly decreased after moxibustion (Figures 3(g)–3(j)).

### 3.4. Moxibustion Affects the Expression of m6A and Its Related Enzymes in Hippocampal and Cortical Areas of APP/PS1 Mice

**Figure 4:**
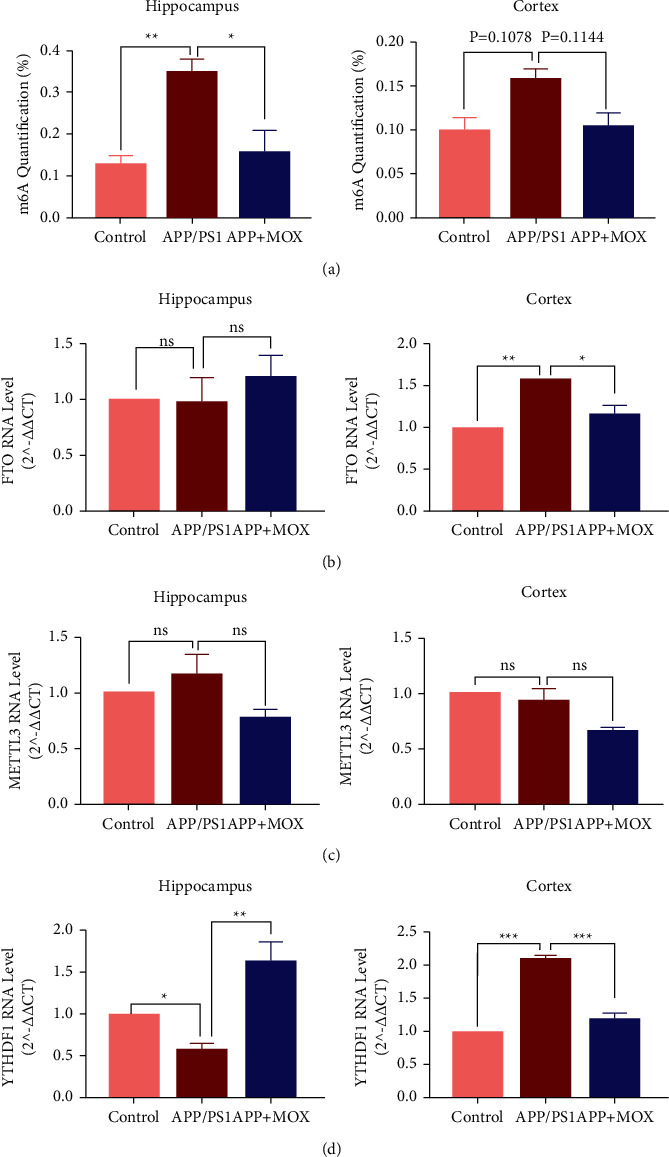
Moxibustion affects the expression of m6A and its related enzymes in hippocampal and cortical regions of APP/PS1 mice. (a) The effect of moxibustion on the total level of m6A in hippocampal and cortical areas. Effects of moxibustion on mRNA expression of FTO (b), METTI3 (c), and YTHDF1 (d) in hippocampal and cortical regions of mice in each group. ^∗^*P* < 0.05, ^∗∗^*P* < 0.01, and ^∗∗∗^*P* < 0.001.

The quantitative detection of m6A RNA methylation results showed that the m6A content in the hippocampal region of mice in the model group was significantly increased compared with that in the control group (*P* < 0.01). After moxibustion treatment, the m6A in mice in the moxibustion group decreased significantly (*P* < 0.05). However, in the cortical region, this difference was not as pronounced as in the hippocampal region (Figure 4(a)).

In addition, we compared the RNA transcript levels of m6A-related enzymes (METTL3, FTO, and YTHDF1) in the hippocampal and cortical regions of mice in each group. Surprisingly, although all three m6A-related enzymes were altered in this model, moxibustion had the most significant effect on YTHDF1 in hippocampal and cortical regions. Compared with the control group, there was no change in FTO RNA in the hippocampus of mice, METTL3 RNA levels tended to increase, and YTHDF1 RNA decreased significantly in the model group (*P* < 0.05). In the moxibustion group, FTO RNA levels tended to increase, METTL3 RNA level tended to decrease, and YTHDF1 RNA levels increased significantly compared with the model group (*P* < 0.01). In the cortical area, compared with the control group, FTO RNA levels increased in the model mice (*P* < 0.01), METTL3 RNA levels did not change significantly, while YTHDF1 RNA levels increased significantly (*P* < 0.001). Compared with the model group, the mice in the moxibustion group showed a significant decrease in FTO RNA levels (*P* < 0.05), a decreasing trend in METTL3 RNA levels, and a significant decrease in YTHDF1 (*P* < 0.001) (Figures 4(b)–4(d)).

### 3.5. Moxibustion Improves the Expression of YTHDF1 in the Hippocampus and Cortical Areas of APP/PS1 Mice

**Figure 5:**
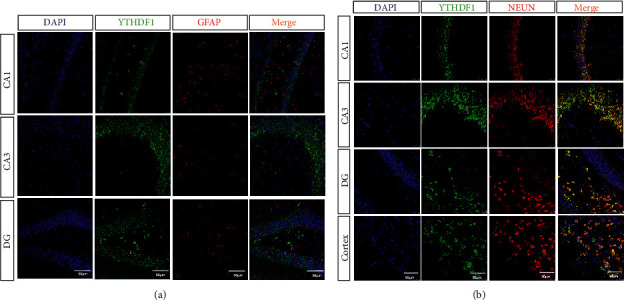
YTHDF1 is mainly expressed in neurons but not in astrocytes. (a) Colabeled immunofluorescence of YTHDF1 and astrocyte marker GFAP in the hippocampus. GFAP (red) labeled astrocytes, YTHDF1 (green), and DAPI (blue). (b) Results of colabeled immunofluorescence of YTHDF1 and the neuronal marker Neun in hippocampal and cortical regions. Neun (red) labeled neurons, YTHDF1 (green), DAPI (blue), and positive coexpression of Neun and YTDF1 (yellow). Magnification in (a–d) = 200×. Scale bars in (a–b) represent 50 *μ*m.

**Figure 6:**
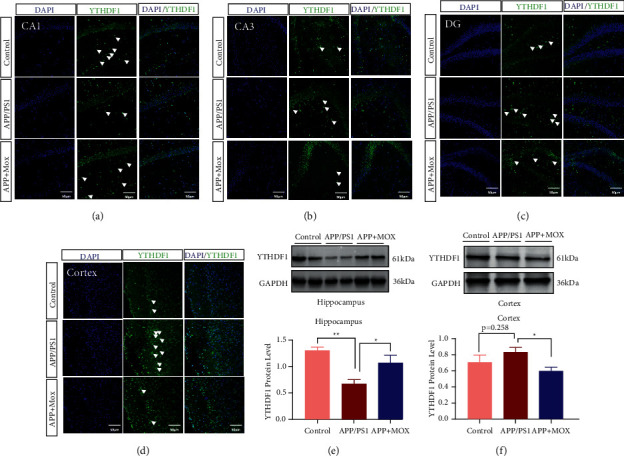
Moxibustion impacts the expression of YTHDF1 in the hippocampus and cortical areas of APP/PS1 mice. Immunofluorescence staining results of hippocampal CA1 (a), CA3 (b), DG (c), and cortical (d) area with FITC 488-conjugated YTHDF1 antibody (green). Protein expression of YTHDF1 in hippocampal (e) and cortex (f) tissues. White arrows indicate the positive expression of YTHDF1. Magnification in (a–d) = 200×. Scale bars in (a–d) represent 50 *μ*m. ^∗^*P* < 0.05 and ^∗∗^*P* < 0.01.

We colabeled YTHDF1 with GFAP or Neun and found that YTHDF1 was mainly expressed in neurons (Figure 5), so we performed immunofluorescence staining for YTHDF1 in hippocampal and cortical regions. However, compared with the control group, the model group mice showed decreased positive expression in the CA1 region of the hippocampus and increased positive expression in the CA3, DG, and cortical regions. Compared with the model group, mice in the moxibustion group showed increased positive expression in the CA1 region of the hippocampus and decreased positive expression in the CA3, DG, and cortical regions (Figures 6(a)–6(d)).

To better illustrate the changes in YTHDF1 expression, we verified the results of qPCR by Western blot. In the hippocampal region, the relative expression of YTHDF1 protein was significantly lower in the model group compared with the control group (*P* < 0.01). The relative expression of YTHDF1 protein was increased in the moxibustion group compared with the model group (*P* < 0.05) (Figure 6(e)). In the cortical area, the amount of YTHDF1 protein in the cortical area of mice in the model group increased compared with the blank control group (*P* < 0.01). After treatment with moxibustion, the amount of YTHDF1 protein in the cortical area of mice in the moxibustion group decreased compared with the model group (*P* < 0.05) (Figure 6(f)).

## 4. Discussion

Many studies have shown that moxibustion has significant advantages in improving the learning and memory ability of AD patients. In addition, our previous studies have shown that 5 days per week for 4 weeks are effective, so in the current study, we chose the same treatment course and found that moxibustion reduced the expression of A*β* and Tau proteins in the hippocampal and cortical regions of APP/PS1 mice.

RNA methylation modifications were discovered by scientists back in the early 1970s [21, 22]. Researchers found that these modifications regulate [23–25] eukaryotic gene expression and can affect various biological processes, such as RNA stability and translation. Conversely, aberrant RNA methylation has been associated with many diseases [26]. RNA methylation includes m6A, m5C, m1A, and m7G, with m6A being the most prevalent and abundant type of RNA methylation modification in eukaryotic mRNAs [27]. A recent study suggests that aberrant m6A methylation may be involved in the process of AD [18]. For instance, it can affect the self-renewal and differentiation of neural stem cells [28, 29]. Since hippocampal neurogenesis occurs at all stages of AD development and decreases as AD progresses, and since hippocampal neurogenesis is also associated with A*β* and Tau proteins [30], we therefore hypothesized that abnormalities in m6A methylation may be associated with the deposition of A*β* and Tau proteins. Besides, there is evidence that oxidative stress in APP/PS1 mice increases at 3 and 5 months of age, with marked A*β* plaques in the cortex from 5 months of age onwards, accompanied by learning and memory impairment [31–33]. Plaque size and number in APP/PS1 mice continued to increase with age [33], suggesting very early neuronal damage and an important role for early intervention to alleviate neurodegenerative disease. Considering the above factors, our finding that the overall level of m6A was elevated in APP/PS1 model mice and decreased after moxibustion in 5-month-old APP/PS1 mice suggests that moxibustion has a preventive effect on AD by reducing m6A levels in APP/PS1 mice.

The function of m6A is reported to be regulated by a combination of methyltransferases called Writer, Eraser, and Reader. The main role of Writer is to catalyze the modification of adenylate on mRNA by m6A, which includes METTL3/14, WTAP, and KIAA1429. Eraser is to demethylase the base that has been modified by m6A, including FTO and ALKHB5. Reader is to recognize the bases that are modified by m6A and further activate the downstream regulatory pathways such as RNA degradation, miRNA processing, etc. [34–36]. METTL3 is a highly conserved protein with its catalytic capacity [37] and is a core component of the m6A methyltransferase subunit. METTL3-mediated RNA m6A modifications have a direct role in regulating hippocampus-dependent long-term memory formation. It has been found that m6A levels change dynamically with age, with low levels of m6A expression throughout embryonic development and a significant increase in adulthood [38]. FTO is one of the first RNA demethylases discovered and plays a vital role in learning and memory. It is reported that FTO deficiency reduces proliferation and neuronal differentiation of adult neural stem cells *in vivo*, leading to impaired learning and memory [39]. Several studies have shown that YTHDF1 is a methyl-binding enzyme that mediates the Wnt/*β*-catenin signaling pathway and may be an important pathway for its involvement in neurodevelopment and neurological diseases. In addition, it has been shown that m6A affects synaptic plasticity [40]. m6A promotes learning and memory through its binding protein YTHDF1 in the adult mouse hippocampus in response to neuronal stimulation and facilitates protein translation of target transcripts. In contrast, specific knockdown of YTHD impairs learning and memory functions. Besides, it also reduces excitatory synaptic function of neurons in the hippocampal region, while synaptic plasticity is disrupted [41, 42], suggesting that memory formation is affected by YTHDF1. Therefore, this study focused on the modulatory effects of moxibustion on the methyltransferase METTL3, demethylase FTO, and methyl-binding enzyme YTHDF1 in APP/PS1 mice. We found that METTL3, FTO, and YTHDF1 were differentially altered in the hippocampal or cortical regions of APP/PS1 mice. However, moxibustion mainly increased the expression of m6A-binding enzyme YTHDF1 in hippocampal and cortical regions of APP/PS1 mice by increasing the expression of m6A-binding enzyme YTHDF1. Moxibustion modulated the expression of METTL3 and FTO, but not significantly, for reasons that are not clear, but we speculate that it may be related to the duration of moxibustion treatment. The next study could extend the treatment duration appropriately. In addition, our results were based on altered protein levels, and more evidence will be provided in future studies.

Taken together, moxibustion improved the learning memory ability and reduced the deposition of A*β* and Tau-like protein pathological products in APP/PS1 mice, which may be related to the fact that moxibustion reduced the total amount of m6A in the hippocampus and cortex of APP/PS1 mice and had a significant effect on the m6A-binding enzyme YTHDF1. However, this study only observed the overall changes of m6A methylation after moxibustion and did not further investigate its function. In addition, the specific pathways that moxibustion regulates m6A methylation have not been clarified. In future studies, the targets of m6A methylation and their regulatory pathways will be investigated.

## Figures and Tables

**Table 1:** Primers used for gene amplification in the q-PCR experiments.

Target gene	Host	Primers (5′∼3′)
YTHDF1	Mouse	F: ACAGTTACCCCTCGATGAGTG
		R: GGTAGTGAGATACGGGATGGGA

FTO	Mouse	F: TTCATGCTGGATGACCTCAATG
		R: GCCAACTGACAGCGTTCTAAG

METTL3	Mouse	F: CTGGGCACTTGGATTTAAGGAA
		R: TGAGAGGTGGTGTAGCAACTT

## Data Availability

The data used to support the findings of this study are included in the article.
